# Understanding the interaction between cytomegalovirus and tuberculosis in children: The way forward

**DOI:** 10.1371/journal.ppat.1010061

**Published:** 2021-12-09

**Authors:** Laura Olbrich, Lisa Stockdale, Robindra Basu Roy, Rinn Song, Luka Cicin-Sain, Elizabeth Whittaker, Andrew J. Prendergast, Helen Fletcher, James A. Seddon

**Affiliations:** 1 Division of Infectious Diseases and Tropical Medicine, Ludwig-Maximilians-Universität München, Munich, Germany; 2 German Center for Infection Research (DZIF), Partner site Munich, Munich, Germany; 3 Oxford Vaccine Group, Department of Paediatrics, University of Oxford, Oxford, United Kingdom; 4 NIHR Oxford Biomedical Research Centre, University of Oxford, Oxford, United Kingdom; 5 The Jenner Institute, The Nuffield Department of Medicine, University of Oxford, Oxford, United Kingdom; 6 Clinical Research Department, London School of Hygiene & Tropical Medicine, London, United Kingdom; 7 Division of Infectious Diseases, Boston Children’s Hospital, Boston, Massachusetts, United States of America; 8 Helmholtz Centre for Infection Research, Braunschweig, Germany; 9 German Centre for Infection Research (DZIF), Partner site Hannover-Braunschweig, Braunschweig, Germany; 10 Department of Infectious Diseases, Imperial College London, London, United Kingdom; 11 Department of Paediatric Infectious Diseases, Imperial College Healthcare NHS Trust, London, United Kingdom; 12 Blizard Institute, Queen Mary University of London, London, United Kingdom; 13 Zvitambo Institute for Maternal and Child Health Research, Harare, Zimbabwe; 14 Department of Infection Biology, London School of Hygiene & Tropical Medicine, London, United Kingdom; 15 Desmond Tutu TB Centre, Department of Paediatrics and Child Health, Stellenbosch University, Cape Town, South Africa; University of Queensland, AUSTRALIA

## Abstract

Over 1 million children develop tuberculosis (TB) each year, with a quarter dying. Multiple factors impact the risk of a child being exposed to *Mycobacterium tuberculosis* (*Mtb*), the risk of progressing to TB disease, and the risk of dying. However, an emerging body of evidence suggests that coinfection with cytomegalovirus (CMV), a ubiquitous herpes virus, impacts the host response to *Mtb*, potentially influencing the probability of disease progression, type of TB disease, performance of TB diagnostics, and disease outcome. It is also likely that infection with *Mtb* impacts CMV pathogenesis. Our current understanding of the burden of these 2 diseases in children, their immunological interactions, and the clinical consequence of coinfection is incomplete. It is also unclear how potential interventions might affect disease progression and outcome for TB or CMV. This article reviews the epidemiological, clinical, and immunological literature on CMV and TB in children and explores how the 2 pathogens interact, while also considering the impact of HIV on this relationship. It outlines areas of research uncertainty and makes practical suggestions as to potential studies that might address these gaps. Current research is hampered by inconsistent definitions, study designs, and laboratory practices, and more consistency and collaboration between researchers would lead to greater clarity. The ambitious targets outlined in the World Health Organization End TB Strategy will only be met through a better understanding of all aspects of child TB, including the substantial impact of coinfections.

## Author summary

Over 1 million children develop tuberculosis (TB) each year, and a quarter of a million children die from this disease. It is increasingly recognised that cytomegalovirus (CMV), a virus that is very common in young children, especially in communities living in poverty, disrupts the immune response to the bacteria that cause TB and increases the chance that a child will develop TB disease. CMV may also change the way that TB manifests in children, how easily TB is diagnosed, and influence the outcome of children with TB. Current research on the interaction between TB and CMV in children is impeded by inconsistent research approaches and laboratory techniques. In this article, we describe what is known about TB, CMV, and about how the 2 pathogens interact in children. We then outline the research gaps that currently exist and make suggestions about the potential studies that might answer these questions. We believe that a better understanding of the interaction between these 2 pathogens could have a substantial impact on child health globally.

## Introduction

It is estimated that over 70 million children are currently infected with *Mycobacterium tuberculosis* (*Mtb*) [1], and, each year, 1.2 million children develop tuberculosis (TB) disease [2]. Of these, a quarter die, representing one of the leading causes of child death globally [1]. Current public health measures to address childhood TB rely mainly on passive case finding, where children who have already developed TB disease present to health systems to be diagnosed and treated. Currently, only about half of the children who develop TB disease are diagnosed [2]. Preventing TB in children would be preferable to waiting for them to become sick.

Although reducing TB exposure is central to preventing childhood TB, approaches that reduce the risk of progression from infection to disease would also be highly impactful. Conditions that are known to affect T-cell immunity, such as HIV, immunosuppressive drugs, and malnutrition, have been shown to increase risk of progression from TB infection to disease [3]. Children are regularly exposed to multiple viral and bacterial pathogens, all of which, to some extent, modulate the developing immune system. It is likely that coinfection with other pathogens can impact host susceptibility to, and ability to contain, *Mtb* [4]. Human cytomegalovirus (CMV) is one of the most immunogenic viruses that infects children [4–6], and CMV in low-resource settings is almost universal in early childhood [7,8]. Emerging epidemiological and immunological evidence suggests that there is a link between CMV infection and progression to TB disease [9–13]. Although CMV is rarely symptomatic in immunocompetent children, infection, reinfection, and reactivation of CMV might have extensive implications for the immunological response to other pathogens, particularly *Mtb*.

A clearer insight into the immunological interaction between these 2 pathogens could have profound impacts on TB vaccine development or host-directed therapies. Given the advanced stage of CMV vaccine development [14,15], it might also be possible to investigate the use of a CMV vaccine to impact TB infection or progression of disease, at least in the youngest children. Understanding the epidemiological and clinical interaction could lead to interventions that might prevent TB or assist in the diagnosis and treatment of TB in children. In this article, we review what is known about the epidemiological, clinical, and immunological interaction between TB and CMV in children and discuss how HIV may impact this relationship. We then outline areas of uncertainty that require further study and make suggestions as to the type of studies that could answer some of the remaining questions. Consensus for these research priorities and future areas for investigation were arrived at through multiple cycles of meetings, conference calls, and written feedback among the authorship who bring expertise in studies of CMV and/or TB in children.

## TB

Individuals with infectious TB disease generate aerosolised *Mtb* that can remain in the air for long periods. Children in close contact with these TB cases are at high risk of breathing in bacilli, and, once bacilli reach the terminal alveoli, they are ingested by macrophages. If macrophages fail to eradicate the mycobacteria, the adaptive immune system is sensitised, a situation that can be detected through a tuberculin skin test (TST) or an interferon gamma release assay (IGRA). If one of these tests is positive, but the child has a normal chest X-ray and has no symptoms, the child is said to have TB infection. If the mycobacteria proliferate, clinical symptoms and signs develop, and the child is said to have TB disease [3].

### Epidemiology of TB infection and disease

The prevalence of TB infection increases with age due to cumulative exposure [16]. The rate of this increase is determined by the force of infection, a reflection of the prevalence of infectious TB in that context. The risk of progressing from TB infection to TB disease however, has a more complex interaction with age and is also influenced by gender. Young children are at high risk of progression from infection to disease, with half of TB-infected infants (<12 months) becoming symptomatic within 12 months. The risk falls rapidly during childhood, with primary school-age children (from around age 5 years until the onset of puberty) being at low risk [17]. As individuals enter adolescence, the risk increases, rising first in girls and then boys [18]. The type of TB that develops is also age dependent. The predominant type of disease in young children is intrathoracic lymph node disease, either simple enlargement or enlargement that leads to complications such as bronchial obstruction or erosion into the lung parenchyma, causing a pneumonic picture. Young children are also more likely to develop severe forms of disseminated disease, such as miliary TB or TB meningitis [19]. As children enter puberty, adult-type disease, with extensive parenchymal involvement and cavities, begin to predominate.

### Immune response to *Mtb*

Upon internalisation of aerosolised droplets of *Mtb* into the airways of a new host, the initial immune response is characterised by an influx of phagocytic cells including resident alveolar macrophages, lung dendritic cells (DCs), and neutrophils [20]. *Mtb* bacilli are taken up by a variety of cell types including DCs, macrophages, neutrophils, monocytes, and epithelial type II pneumocytes [21]. Infected DCs migrate to the local draining lymph node 8 to 12 days after infection where they activate antigen-specific T cells and drive differentiation towards an inflammatory Th1 phenotype [22]. Despite human observational studies and experimental animal models showing that the Th1 cytokines interferon (IFN)-γ, tumour necrosis factor (TNF)-α, and interleukin (IL)-12 are critical in protection against acquisition of infection and progression to TB disease [23,24], these factors alone do not explain the large heterogeneity in clinical outcomes. More likely is that disease outcome is dependent on many host, pathogen, and environmental interactions [22].

### Immune evasion strategies of TB

*Mtb* has evolved a variety of immune evasion strategies that have been thoroughly reviewed elsewhere [25]. Some examples include (1) the capacity of *Mtb* to block phagosome–lysosome fusion by secretion of a lipid phosphatase, SapM, which hydrolyzes phosphatidylinositol 3-phosphate PI3P, a host membrane trafficking regulatory lipid essential for phagosomal maturation and phagosome–lysosome fusion [26]; (2) induction of macrophage production of the immune regulatory cytokine IL-10, which prevents phagosome maturation and phagolysosomal fusion [22]; (3) ESAT-6 directed sequestration of host beta-2-microglobulin resulting in the down-regulation of macrophage antigen presentation through MHC Class-I [27]; (4) lipoarabinomannan (LAM) signalling through alveolar macrophage mannose receptor to reduce the cellular secretion of pro-inflammatory cytokines TNF-α and IL-1β and chemokines MCP-1 and IP-10, thereby impairing recruitment of innate immune cells to the lungs [28,29]; and (5) delaying migration of antigen-presenting cells (APCs) to the draining lymph node by poorly understood mechanisms (by an estimated 3 weeks), resulting in subsequent delays in T-cell priming [30].

**Fig 1:**
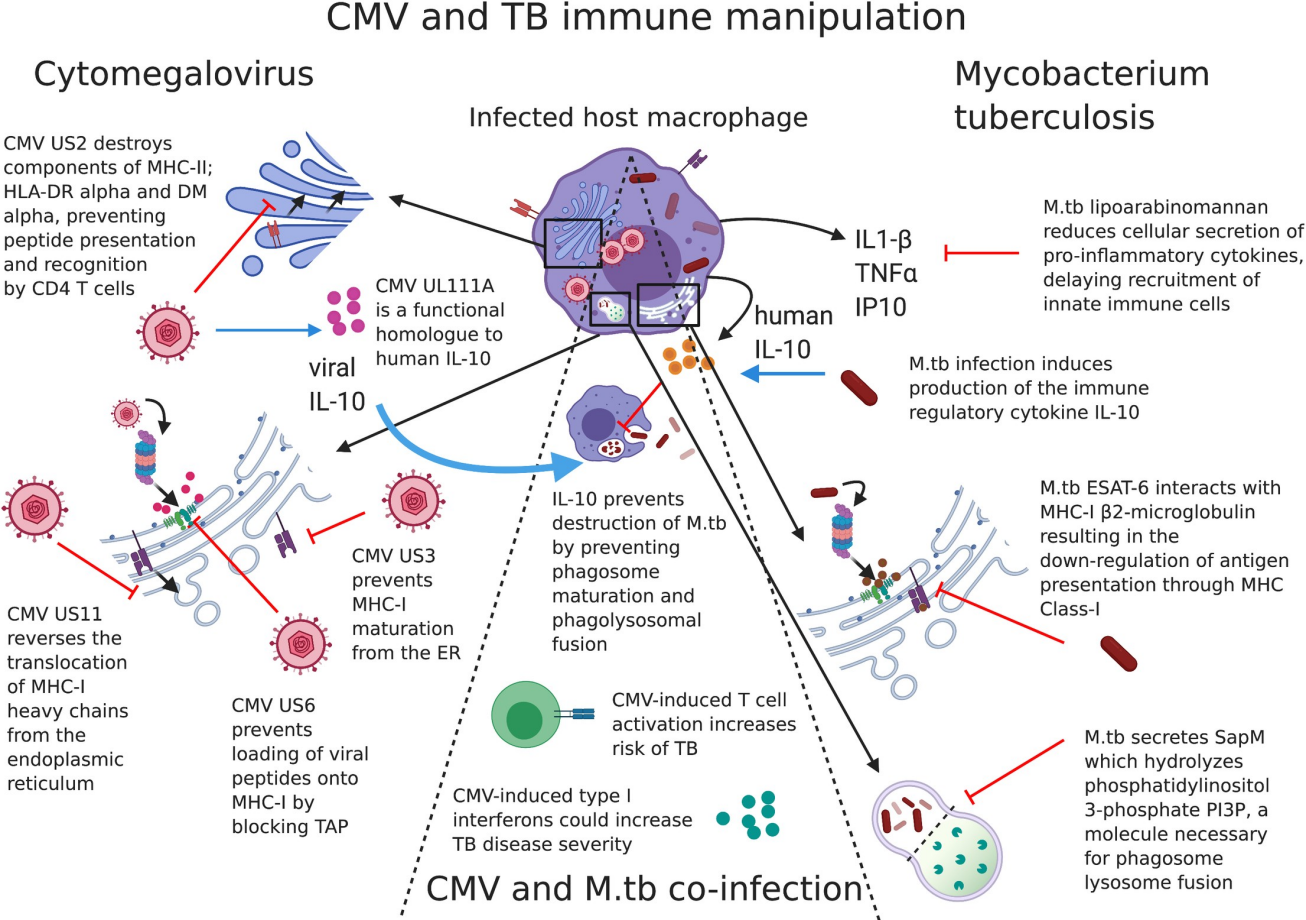
CMV and TB immune mechanisms of host manipulation. Immune manipulation by CMV (left-hand side), *Mtb* (right-hand side), and some examples of possible interactions between the 2 pathogens (centre) in a host cell. Examples of viral genes expressed during both active replication and latent infection aid CMV in avoiding viral peptides to be displayed on the surface of infected cells. Mtb manipulates cellular machinery to increase or decrease cytokine production and Mtb protein early secreted antigen (ESAT6) down-regulates presentation of mycobacterial peptides via MHC-I. CMV-derived viral IL-10 may interfere with protection against Mtb, and CMV-induced immune activation and enhanced type-I IFNs could increase risk and severity of TB disease. While interactions between CMV and Mtb may occur directly while a host cell is infected with both pathogens, alterations to the wider immunological environment by either pathogen may provide conditions conducive to pathogenesis by the other. The example of immune manipulation mechanisms shown here is not an exhaustive list. CMV, cytomegalovirus; ER, endoplasmic reticulum; IFN, interferon; IL, interleukin; MHC, xxx; *Mtb*, *Mycobacterium tuberculosis*; TAP, xxx; TB, tuberculosis; TNF, tumor necrosis factor.

In addition, the bacteria themselves have evolved ways of manipulating the ability of the host to produce IFN-γ. The early secreted proteins ESAT-6 and CFP-10, which form 2 of the gene products of a 9.5-kb section of *Mtb* DNA called Region of Deletion 1 (RD1), are involved in virulence and pathogenesis of *Mtb* [31]. ESAT-6 contributes to virulence by inhibiting T-cell IFN-γ production [32]. An overview of some of these strategies is detailed in **Fig 1**.

## CMV

### Epidemiology of CMV infection and disease

Congenital CMV infection, where the virus is transmitted in utero, is a leading cause of permanent hearing loss and neurological impairment as well as vision loss in infants worldwide [33]. Maternal primary infection or reactivation, especially during the first trimester of pregnancy, is particularly associated with adverse neonatal outcomes [34]. The incidence of congenital CMV infection is estimated at between 0.7% and 5% of all births in low- and middle-income countries (LMICs [35]) and approximately 0.3% of births in the United Kingdom [36].

Postnatal CMV acquisition occurs predominantly through breastfeeding and secretions (e.g., saliva and urine) from infected mothers and siblings. CMV acquired after birth is usually mild and often asymptomatic; however, the virus establishes lifelong latency, with intermittent viral reactivation across the life course, which may result in severe complications in immunocompromised hosts, such as transplant recipients [37].

The age of postnatal CMV acquisition varies greatly geographically. In LMICs, the prevalence of CMV infection is extremely high in childhood and probably universal in infancy, an important point in light of the risk of progression from TB infection to disease in this age. In a study in Gambia, 86% of children were infected by the age of 12 months [38]; similar findings were reported from Uganda, where 95% had immunological evidence of a CMV infection by the age 5 [11]. By contrast, in the UK, 15% of 1 to 4 year olds, 30% of 20 to 29 year olds, and approximately 80% of the population was infected by age 65 [39]. Prevalence surveys in other high-income countries demonstrate a progressive increase in seropositivity with age, with females consistently having higher rates of seropositivity compared to males in all age groups [40–42].

Data from the United States show that socioeconomic status is a strong predictor of latent infection with CMV and other herpesviruses [43].

### Immune response to CMV

The immune response to CMV infection is one of the largest ever documented, marked by persistence of terminally differentiated antigen-specific T cells [44]. Maintenance of CMV in a latent state is therefore a very resource-intensive activity for the immune system. Characterisation of the CMV-specific T-cell population revealed that up to 30% of circulating CD4+ and CD8+ memory compartments are dedicated to the restraint of viral replication in CMV seropositive individuals [44], and these percentages may go even higher in the very old host [45]. This phenomenon, termed “memory inflation,” is linked to permanently high expression of IFN-γ and other Th1 cytokines to induce a chronic pro-inflammatory state associated with immune activation [46] and has been found to correlate with acute phase response proteins such as C-reactive protein (CRP) [46,47]. While early findings argued that CMV infection may accelerate natural immune ageing processes [48], subsequent studies showed a more nuanced picture [49,50]. CMV infection imprints the immune system [51,52], but its effects on the immune response to vaccines or protection upon experimental infection are rather modest [53,54]. On the other hand, the immunopathology of chronic inflammation reflects an association of CMV infection with frailty and increased mortality in the elderly, especially those belonging to lower socioeconomic groups [55–58]. CMV infection is also linked to long-term cardiovascular disease [59,60] or diabetes mortality [61]. A study of 105 twin pairs, which measured over 200 cellular and serum characteristics, found that most of the differences in immune parameters were due to nonheritable factors. The authors described that discordant CMV infection status in monozygotic twins was found to be associated with differences in 58% of all parameters tested [62]. At the other end of the age spectrum, it has been shown that CMV infection in infants leads to profound immune dysfunction, specifically differentiation of the CD8 compartment [4].

While the exact sites of latency are incompletely understood, myeloid lineage cells, including monocytes, are well known to harbour silenced CMV genomes during latency [63,64]. Monocytes are not permissive to viral replication, but CMV infection can drive cell differentiation to macrophages [65], which do support the full life cycle of CMV. CMV has a very large (230 kb) genome, and a multitude of viral genes, expressed during both active and latent CMV infection, interfere with both innate and adaptive immune responses [66]. These viral genes will not be reviewed in detail here, but some examples are described in **Fig 1** and include genes that prevent antigen presentation on human leukocyte antigen (HLA) molecules, subvert natural killer (NK) cell recognition of infected cells [66], or avoid innate antiviral effects by myeloid cells [67]. For instance, UL111a, a functional homologue to human IL-10 (hIL-10) [68] binds with high affinity to the hIL-10 receptor despite its low structural homology to hIL-10.

### TB–CMV interaction

Spatial and temporal similarities between TB and CMV underscore the biological plausibility of a hypothetical interaction. Both pathogens infect the same cell types in the same organ, and both can establish chronic latency in these cells in the lung [69–71]. The very similar age–sex distribution of the 2 pathogens and the shared risk factors add credence to the idea of an immunological link between CMV and TB [9]. The interaction between viruses and bacteria is not a new phenomenon [72]: The associations between HIV and TB have been well documented, and CMV-associated symptoms, such as retinitis, constitute AIDS-defining illnesses [1,73,74]. Severe bacterial pneumonia is common following influenza in human populations [75], and, experimentally, it has been found that mycobacterial growth is enhanced, and survival is decreased, when mice are exposed to influenza A virus prior to *Mtb*, in a type I IFN-dependent pathway [76].

Type I IFNs are key components in antiviral immunity. Syntheses of IFN-α and IFN-β are rapidly induced after exposure of host cells to CMV [77]. Many viruses have evolved ways to suppress the antiviral activity of type I IFNs and the 72-kDa IE1 protein (IE1-72 kDa) of CMV confers partial resistance to these antiviral cytokines [78]. Evidence of both the deleterious and putative protective role of type I IFNs in TB disease is reviewed by Moreira-Teixeira and colleagues [79]. In humans and in mouse models, excessive type I IFNs are also linked to TB disease exacerbation via an eicosanoid imbalance, whereby necrotic, as opposed to apoptotic, cell death is induced, resulting in subsequent bacterial escape and further cellular infection [80]. Some examples of overlapping immune manipulation mechanisms of TB and CMV are shown in **Fig 1**.

It is important to consider the link between HIV and TB, and HIV and CMV, and the very likely 3-way interaction between CMV, TB, and HIV [3]. The targeted depletion of TB-specific CD4+ T cells by HIV [81] highlights the importance of this cell type in particular in the immunological response to TB. T-cell activation and associated pro-inflammatory immune state due to CMV infection have been demonstrated in people living with HIV [82,83], and repeated exposure to *Mtb* is linked to an augmented activation of T cells [84]. Both these processes likely lead to HIV disease progression through T-cell activation [85]. CMV is known to imbalance systemic cytokine, T-cell, and macrophage responses [86], and inflammation caused by either (re)infection or reactivation could lead to an increased risk of infection with TB or progression to TB disease [9]. Mouse models suggest that CMV-associated immunosenescence and subsequently impaired responses to heterologous infections may be CMV dose dependent [87].

**Fig 2:**
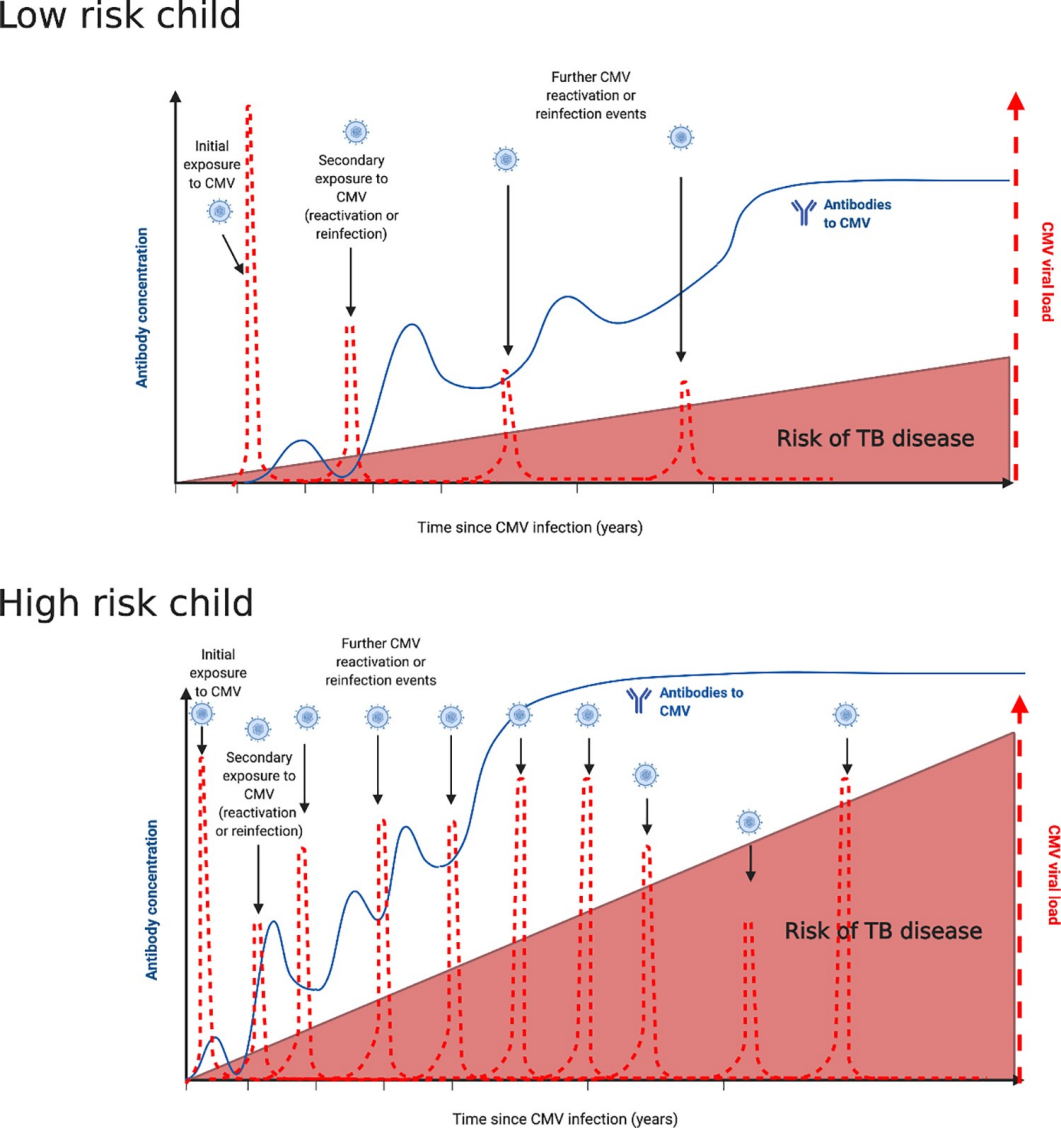
Conceptual framework of TB and CMV interaction in low- and high-risk children. Conceptual framework of TB and CMV interaction in high-and low-risk children. Black arrows represent CMV infection, reactivation, or reinfection events. Red dashed lines show hypothesised CMV viral load following infection, reactivation, or reinfection events. Blue line represents IgG antibody levels to CMV. Lower risk child—immune competent children aged approximately between 4 and 15. Higher risk children—children aged less than 4 years old, immune compromised, HIV infected, older adolescent (over 15 years of age). Created with BioRender.com. CMV, cytomegalovirus; TB, tuberculosis.

**Table 1** summarises the literature in which CMV and TB coinfection has been investigated; much of the work to date has been done in adults. In a Phase 2b clinical trial of a developmental TB vaccine, immune activation, characterised by increased HLA-DR on CD4+ T cells, was associated with increased risk of TB disease in South African infants [12]. As the major driver of immune variation [62] and a cause of T-cell activation, CMV associations were investigated, and a positive correlation was found between a CMV-specific IFN-γ response and CD8+ T-cell activation. In the same infant cohort, and in an adolescent cohort, this CMV profile was associated with an increased risk of developing TB disease and shorter time to TB diagnosis [13]. Increased serum IgG to CMV in Ugandan individuals (aged 3 to 56 years) was associated with increased risk of symptomatic TB disease (odds ratio 2.8 for medium and 3.5 for high IgG levels) [10], while increased IgG responses to the herpes viruses Herpes Simplex (HSV1/2) and Epstein–Barr virus (EBV) were not associated with any increased risk of TB disease in the same cohort [10]. It is hypothesised that repeated exposure (either reactivation or reinfection) could lead to increased IgG levels (**Fig 2**). More mechanistic studies are required to determine if CMV-related changes in the immune system observed in TB are due to an increased replication of CMV itself or are a manifestation of an altered immune response at large. Investigation of total IgG levels, which are indicative of nonspecific B cell activation, were not associated with CMV IgG levels across over 2,000 individuals in rural Uganda [88], nor were they correlated with anti-CMV antibodies among nontuberculous mycobacteria patients or controls in a study conducted in Australia [89].

**Table 1:** Studies that have evaluated the interaction between TB and CMV.

Author, year	Population	Location	Type of study	Findings
Olaleye, 1990 [90]	Adult TB patients	Nigeria	Cross-sectional serological study of CMV prevalence among 161 TB patients, 89 patients other than TB, and 110 healthy donors	Complement fixing antibodies to CMV were higher among TB patients compared with non-TB hospital patients and healthy controls
Nagu, 2017 [91]	Adult PTB patients	Tanzania	Cross-sectional study of cellular IFN-γ responses to CMV and EBV antigens among 234 TB patients, 213 who survived, and 21 who died at end of treatment	PBMCs from patients who survived (after treatment completion) exhibited significantly stronger IFN-γ responses to CMV (*p* = 0.035), EBV (*p* = 0.006) or *Mtb* ESAT-6 (*p* = 0.043) at the time of diagnosis as compared to patients who died during TB treatment. Moreover, 71% of patients who died were HIV positive, whereas 38% of those who survived were HIV positive. Analyses were not adjusted for HIV infection. Immune responses may be an indicator of general immune fitness and may have been an independent predictor of survival
Amran, 2016 [89]	NTM patients and healthy controls	Australia	Cross-sectional serological study among 112 pulmonary NTM patients and 117 controls	Elevated levels of CMV antibodies were found in plasma from patients with pulmonary NTM disease. Exclusion criteria included HIV infection, excessive alcohol consumption and smoking. Total IgG levels were investigated with no association with anti-CMV antibody levels
Sirenko, 2003 [92]	Children and adolescents	Russia	Cross-sectional study of 65 children and adolescents with respiratory TB	TB cases were 3 times as likely to be infected with CMV than non-TB individuals. Severity of TB disease was associated with increased CMV antibody levels compared with mild cases of TB
Fletcher, 2016 [12]	Infants and adolescents	South Africa	Nested case–control study from a Phase 2b efficacy study of TB vaccine candidate MVA85A. Study included 53 TB case infants and 205 matched controls. Independent adolescent cohort used to verify findings	Association of activated HLA-DR+ CD4+ T cells and risk of TB disease. Positive correlation between T-cell activation and CMV IFN-γ response
Muller, 2019 [13]	Infants and adolescents	South Africa	Cellular IFN-γ responses to CMV antigens. Same population as [12]	A CMV-specific IFN-γ response was associated with CD8+ T-cell activation and increased risk of developing TB disease and shorter time to TB diagnosis
Stockdale, 2018 [11]	All ages PTB	Uganda	Cross-sectional serological study of CMV IgG levels in 2,174 individuals in rural Uganda, 27 PTB cases	CMV seropositivity was 83% by 1 year of age, increasing to 95% by 5 years. Female sex, HIV positivity and PTB were associated with an increase in CMV IgG levels in adjusted analyses
Stockdale, 2019 [88]	All ages PTB	Uganda	Cross-sectional serological study of CMV IgG levels in 2,189 individuals in rural Uganda, 27 PTB cases. Same population as [11]	Higher CMV IgG levels (used as a measure of CMV exposure) were associated with lower levels of some antimycobacterial antibodies, but no increase in total IgG. HIV infection was associated with a decrease in all antimycobacterial antibodies measured and with an increase in total IgG. Analyses were adjusted for age and sex
Stockdale, 2020 [93]	All ages	Uganda	Nested case–control study (nested within [88]) of 25 PTB patients up to 10 years prior to TB diagnosis with 3 to 6 matched controls per case	IgG response to CMV, but not Epstein–Barr or herpes simplex virus, was associated with increased risk of active TB disease up to 10 years before diagnosis. Individuals with medium anti-CMV IgG were 2.8 times more likely to have PTB (*p* = 0.055), and those with high anti-CMV IgG 3.4 times more likely to have PTB (*p* = 0.007). Mycobacterial antibody levels were not associated with differences in odds of PTB disease. Nonspecific B cell activation (as measured by total IgG levels) was not associated with CMV IgG response

CMV, cytomegalovirus; EBV, Epstein–Barr virus; IFN, interferon; PBMC, peripheral blood mononuclear cells; PTB, pulmonary tuberculosis; NTM, nontuberculous mycobacteria; TB, tuberculosis.

## Knowledge gaps and how to address them

While CMV may play an important role in the pathogenesis of TB, major areas of uncertainty exist. In this section, we will highlight specific gaps in the literature and systematic approaches researchers might use to ensure future studies are efficient and comparable.

### Understanding the burden

Little evidence is available concerning the true burden of TB–CMV coinfections. Similar age, sex, and socioeconomic status distributions exist for both diseases, and they share remarkably comparable risk factors. Quantifying the prevalence of TB–CMV coinfection on a population level, by age group, geographical region, and with or without HIV, is crucial to identify risk factors for infection, disease progression, and poor outcome. As detailed in **Table 2**, this could be achieved through cross-sectional surveys and investigation of longitudinal cohorts (ideally nested within existing cohorts where TB infection and disease status is already characterised). The latter is especially valuable if regular sampling is included to understand the timing of CMV and TB infection, as it remains difficult to elucidate which pathogen came first in coinfection and what impact that may have. These study designs might include paediatric household contact studies with a case–control design (CMV positive versus CMV negative), long-term population-based cohort studies, such as birth cohorts, and vaccine studies.

**Table 2:** Understanding the burden for CMV–TB interaction.

Question	Knowledge gap (summary)	Potential study designs	Parameters and samples to be evaluated
How prevalent are CMV–TB coinfections, and what are risk factors for these infections?	- Quantifying the burden, timing, and outcome of TB–CMV coinfections across different sites and in different risk groups- Impact of co-factors on CMV–TB prevalence- Identifying individual risk factors associated with TB–CMV coinfection- Identifying individual characteristics associated with poor outcome	- Systematic review of existing literature- Cross-sectional and longitudinal cohorts (observational, diagnostic, and randomised intervention studies in humans nested within well-characterised TB cohorts) to quantify prevalence and risk factors for CMV–TB coinfection- Modelling studies to evaluate the number of deaths from TB–CMV coinfections, given number of cases, the proportion diagnosed, and expected mortality treated and untreated	Acute versus latent infection (CMV)- Viral detection: whole blood (EDTA) for viral load (PCR), respiratory specimen, and others- Serology (plasma/serum): quantitative IgG, IgM, and IgG avidity TB diagnostic workup:- TB microbiology- Immunoassays evaluating T-cell response- Novel biomarkers in blood and urine
How do TB, CMV, and HIV interact?	- Impact of HIV on prevalence of CMV–TB coinfections and disease course- Impact of CMV in HIV-infected on TB progression and clinical presentation- Impact of CMV–TB coinfection on course of HIV	- In vitro models including isolated cell populations and mechanistic models- Observational, diagnostic, and randomised intervention studies in humans- In vivo models including mouse and nonhuman primate coinfection- Longitudinal cohorts from varying geographical areas and with different patient populations	Acute versus latent infection (CMV)- Viral detection: whole blood (EDTA) for viral load (PCR), respiratory specimen, and others- Serology (plasma/serum): quantitative IgG, IgM, and IgG avidity- Evidence for exacerbation of TB disease or activation of latent TB infection in CMV/MTB coinfected animals TB diagnostic workup:- TB microbiology- Immunoassays evaluating T-cell response- Novel biomarkers in blood and urine HIV- Viral detection: whole blood (PCR)- CD4 and CD8 T-cell count (absolute, %)

CMV, cytomegalovirus; PCR, polymerase chain reaction; TB, tuberculosis.

Diagnostic tools need to be standardised and systematically applied, to allow consistent reporting across studies. This would include the types of samples taken, sample storage and laboratory testing, and would include CMV quantitative viraemia (cell-associated and in serum to attempt to better understand possible tissue-specific compartmentalisation of CMV), CMV quantitative serology, and serological avidity. Ideally, the assays used to determine CMV burden would be able to discriminate between primary CMV infection and reactivation of latent CMV as it is possible that these different forms of CMV could have different impacts on TB disease progression. For TB diagnostics, improved approaches for both TB infection and TB disease are required. For TB infection, more advanced assays for pathogen detection, in addition to immunological sensitisation are needed [94,95], as are tools to determine viable bacilli that are likely to cause future disease [96,97]. For TB disease, microbiological evaluations using traditional respiratory specimens require optimisation, and newer diagnostics, using biomarkers in blood and urine, should be applied [98,99]. Finally, modelling studies could help to estimate burden and mortality from TB–CMV coinfections, given the number of cases, the proportion diagnosed, and expected mortality in treated and untreated individuals. Effect sizes can be used to not only quantify the effect of CMV infection on TB, but also estimate the impact of a CMV vaccine on TB incidence.

A previous study, from a predominantly adult cohort, shows that the odds of progressing to TB disease in individuals with medium (of 3 levels) and high CMV IgG levels were 1.8 and 3.4, compared to those with low CMV IgG levels [10,93]. To understand the excess risk attributed to CMV (or the percentage of TB cases that could be averted if CMV exposure were reduced/removed from the population), we calculated the population attributable fraction (also called the risk difference or excess risk) [100]. To derive a rough estimate of population attributable fractions for having raised CMV IgG levels, odds ratio data from Stockdale and colleagues [93] and the equation pc(ψ – 1)/ψ were used, where pc is the proportion of risk factor among cases only, and ψ is the odds ratio [101]. With the caveats that these estimates are from limited data and are not in a paediatric setting, among rural Ugandan individuals with a medium level (of 3 levels) of CMV IgG as measured by ELISA (1.04 to 1.34 OD units), 25% of the TB cases in that group could be attributed to having a raised level of CMV IgG. Among individuals with high CMV IgG levels (1.35 to 2.84 OD units), 32% of the TB cases in that group could be attributed to having a raised level of CMV IgG. The study used here is cross-sectional, and multiple covariants may have influenced the apparent associations. Additionally, more work is needed to tease apart the relationship between CMV IgG levels, extent of CMV infection, CMV infection that could be avoided by giving a CMV vaccine, and the possible ramifications of that on TB infection and disease progression.

Another possibility is that CMV is impacting upon the protective effect of BCG, the only available TB vaccine. It remains difficult to estimate if, and how, CMV might affect the response to a TB vaccine, as we do not have a clear correlate of protection for TB, neither vaccine induced nor acquired via infection. Effects of CMV on other vaccine responses have been described previously but remain unclear. For influenza vaccine, both up- and down-regulation of immune responses vaccines have been described [102,103], and the response to an Ebola vaccine is pronouncedly reduced in CMV–positive adults [104]. How this might translate to a TB vaccine remains unclear; any modelling would need to be informed by prospectively collected data to derive realistic models.

### Understanding the underlying pathogenesis

The limited number of studies investigating both CMV and TB together point towards overlapping epidemiology and analogous risk factors. Equally, mechanistic studies of each pathogen individually present a sound biologic hypothesis for an interaction. There remains a major gap in our understanding of the underlying pathophysiology, and very little has been published in this field (**Table 1**). However, a recent study has demonstrated that CMV-associated immune activation may play an important role in the pathogenesis of TB in children [12]; immune activation, characterised by increased HLA-DR on CD4+ T-cells, was associated with increased risk of TB disease in South African infants [12,13].

As detailed in **Table 3**, we suggest that studies could be undertaken, assessing mechanisms by which either CMV or TB could exacerbate the other, taking into consideration the impact of TB disease on CMV reactivation, and the impact of CMV on response to TB infection, disease progression, and response to treatment.

**Table 3:** Understanding the underlying pathogenesis and immunology of CMV–TB interaction.

Question	Knowledge gap (summary)	Potential study designs	Parameters and samples to be evaluated
Does CMV impact the host response to *Mtb*, and, if so, by which mechanisms?	- Mechanisms through which CMV impacts acquisition of, and progression to, TB disease (or vice versa)- Evaluation of effects of one infection on the immune response to the other (direct versus indirect)- Mycobacterial or viral characteristics: impact of different strain types of *Mtb*/CMV on disease pathogenesis- Role of CMV reactivation during TB disease and its effect on the response to TB treatment	- In vitro models including isolated cell populations and mechanistic models- Mechanistic models based on both animal models and human specimens from affected populations to study underlying mechanisms, but also as a tool for evaluation of further hypothesis- Immunological studies characterising immune response (esp. T-cell response and activation; host omics—transcriptomics, proteomics, and metabolomics)	Animal model- Dynamics of T-cell and antibody responses specific for CMV and MTB in coinfected animals- Quantification of CMV and MTB in lung, spleen, lymph nodes, and other tissues of coinfected animals- Impact of CMV on myeloid inflammatory responses in animal models Human model/specimen (for TB and CMV):- T-cell response and activation; whole blood/PBMCs- Host omics (transcriptomics, proteomics, and metabolomics); whole blood Acute versus latent infection (CMV)- Viral detection: whole blood (EDTA) for viral load (PCR), respiratory specimen and others- Serology (plasma/serum): IgG, IgM TB diagnostic workup:- TB microbiology- Immunoassays evaluating T-cell response
Does CMV impact the natural history and pathogenesis of TB?	- Correlation between CMV DNA and progression to TB disease- Impact of relative timing of CMV and TB infection on disease progression- Description of relative risk of TB progression in CMV–seropositive children	- Observational studies in humans- Longitudinal cohorts from varying geographical areas and with different patient populations to characterise which patients develop CMV–TB coinfections (risk factors)	Acute versus latent infection (CMV)- Viral detection: whole blood (EDTA) for viral load (PCR), respiratory specimen and others- Serology (plasma/serum): IgG, IgM TB diagnostic workup:- TB microbiology- Immunoassays evaluating T-cell response

CMV, cytomegalovirus; PCR, polymerase chain reaction; TB, tuberculosis.

In addition to clinical investigations, animal and in vitro coinfection models could be important to define early stages of infection and interaction and to highlight mechanistic relationships. There are no established animal models to study CMV–TB interaction to date. The major animal models used for *Mtb* are the mouse, guinea pig, and nonhuman primate, although a wide range of models have been used for TB including zebra fish, rabbit, rats, mini pigs, and cattle [105].

In the case of CMV, the guinea pig (GPCMV) or rhesus macaque (RhCMV) CMV models are most commonly used [106]. Human CMV is host restricted and typically does not infect animal tissue; therefore, rodent-specific CMV strains are used to investigate the pathogenesis of CMV in mouse, rat, and guinea pig, and primate-specific strains are used to investigate infection in rhesus macaques [107,108]. Although CMV infections are highly species specific, human CMV is closely related to the CMV seen in nonhuman primates with a high sequence homology of approximately 97% [106,109,110], making the rhesus macaque a good candidate for an animal model in which TB–CMV coinfection could be studied. However, as RhCMV circulates naturally in nonhuman primate (NHP) colonies, natural exposure at birth can confound infection studies with RhCMV. Therefore, as the mouse model has been used extensively to investigate both *Mtb* and murine cytomegalovirus (MCMV) separately it might have utility for the early investigation of the interactions between TB and CMV before progression to larger animal models. The advantage of the mouse model for exploring TB–CMV coinfection is the tractability of this model for exploration of immune mechanisms with the wide availability of different strains, gene knockouts, and immune reagents. There are standardised mouse models for the exploration of *Mtb* infection, and the mouse model is routinely used for assessing TB vaccine and drug efficacy [111]. Although MCMV infection does not mirror the clinical aspects of human CMV infection, there are parallels, in particular the strong and sustained immune activation and CD8+ T-cell memory inflation driven by the MCMV Smith strain in the mouse, which is a parallel of the inflated HCMV memory response in humans [112]. Also, in parallel with human CMV, the lung in mice is a reservoir of latent MCMV infection and frequently the site of viral reactivation, which drives inflammation and can cause pulmonary fibrosis [113]. Mouse models of TB–CMV will be of particular value when questions are focused on clinical and immunological observations from human studies. Examples of human in vitro models are outlined in **Table 3**.

### Understanding clinical impact

Primary CMV infection and reactivation are often linked to poor long-term health outcomes. In bacterial sepsis, CMV reactivation (similar to other herpesviruses) is associated with worse clinical outcomes (including mortality) as well as longer duration of mechanical ventilation [114–116]. For individuals with HIV, simultaneous CMV infection has been shown to impact HIV disease progression and severity of disease, and an independent correlation between CMV DNA viral load and AIDS-defining events has been described [117,118]. In addition, the age-adjusted relative risk of progression to AIDS was 2.5 times higher in CMV–seropositive compared to CMV–seronegative individuals [119].

In **Table 4**, we highlight the importance of determining the direct relationship between CMV infection and severity of clinical presentation of TB, in addition to the possible relationship between CMV infection and other TB risk factors such as malnutrition or HIV. By characterising the coinfection status of CMV, TB, and HIV in clinical cohorts, it would be possible to determine whether CMV is a predictor for mortality or morbidity in TB.

**Table 4:** Understanding the clinical impact of CMV–TB interaction.

Question	Knowledge gap (summary)	Potential study designs	Parameters and samples to be evaluated
Does CMV impact the severity of childhood TB?	Morbidity:- Frequency of severe clinical presentation in CMV–positive versus CMV–negative children- Association of CMV positivity with other morbidities that influence TB presentation (HIV and malnutrition) Mortality:- Identifying individual characteristics associated with poor outcome- Quantify the burden and outcome of TB–CMV coinfections across different sites and in different risk groups- CMV as predictor for mortality in TB	- Systematic review of existing literature- Autopsy studies of deaths from clinical TB/pulmonary infections, etc.- Observational, diagnostic, and randomised intervention studies in humans- Longitudinal cohorts from varying geographical areas and with different patient populations to characterise which patients develop CMV–TB coinfections (risk factors)- Using biobanked clinical samples from TB cohorts	Acute versus latent infection (CMV)- Viral detection: Whole blood (EDTA) for viral load (PCR), respiratory specimen and others- Serology (plasma/serum): IgG, IgM TB diagnostic workup:- TB microbiology- Immunoassays evaluating T-cell response Human model/specimen (for TB and CMV):- T-cell response and activation; whole blood/PBMCs- Host omics (transcriptomics, proteomics, and metabolomics); whole blood
How does CMV affect the way child TB is diagnosed?	- Reliable and feasible reference standards for both CMV and TB- Impact of CMV on disease presentation and diagnosis of children with TB- Diagnostics needed: • Pathogen-based (nuclei amplification, antigen, amd metabolites) • Host response based (host-derived molecules) • Clinical algorithms • Diagnostic strategies	- Observational, diagnostic, and randomised intervention studies in humans • New tests and testing approaches for CMV and TB evaluated in longitudinal cohorts from varying geographical areas and with different patient populations • Adequately designed and powered STARD [120,121] compliant multi-centre diagnostic evaluations• Biobanking of well-characterised samples to aid discovery and evaluation of novel diagnostics	Acute versus latent infection (CMV)- Viral detection: Whole blood (EDTA) for viral load (PCR), respiratory specimen and others- Serology (plasma/serum): IgG, IgM TB diagnostic workup:- TB microbiology- Immunoassays evaluating T-cell response Human model/specimen (for TB and CMV):- T-cell response and activation; whole blood/PBMCs- Host omics (transcriptomics, proteomics, and metabolomics); whole blood

CMV, cytomegalovirus; PBMC, peripheral blood mononuclear cell; PCR, polymerase chain reaction; TB, tuberculosis.

For CMV, there are few tools to differentiate between primary infection, reactivation, and recent reinfection (with the same or a new CMV strain). Stages of disease in TB pathogenesis are similarly difficult to classify, particularly in the absence of reliable diagnostic tools. In **Table 4**, we recommend the systematic measurement of both CMV viral load and serology (IgG, IgM, and IgG avidity) ideally in a range of stored samples from well-characterised TB cohorts to increase the comparability between studies. Use of biobanked samples from well-characterised cohorts could also facilitate the discovery of new and reliable tools and testing approaches to study coinfections and might be an efficient initial approach.

### Interventions

Ultimately, if a link is found, the research outlined in this article should facilitate the identification, evaluation, and implementation of effective interventions. A number of CMV vaccines are currently under evaluation, targeting the prevention of congenital CMV or post-transplant infection (recently reviewed by Plotkin and colleagues [15]). While there are no Phase III data available yet, early evidence from Phase II studies suggest that a vaccine could reduce CMV infection in seronegative individuals by 43% to 50%, compared to placebo groups [15]. In addition, several interventions requiring cultural rather than programmatic changes have been described, such as parenting practices or handwashing to reduce or at least delay CMV transmission [122–124]. Another area that that might lead to substantial impact is in the field of biomarkers to predict future TB disease progression. If studies can identify easily measurable CMV-associated predictive signatures of TB progression, interventions could be targeted at high-risk children.

Interventions or programmatic changes, ideally optimising and interlinking current programmes on TB/CMV control and outcome, could be either targeted at populations at large, or at specific subgroups, such as HIV–positive children with chronic lung disease (**Table 5**). As CMV is ubiquitous, the aim of those would not necessarily be to prevent, but rather delay, infection. If CMV infection increases the risk of acquisition of primary TB infection or progression from infection to disease, then a delay in CMV infection might have an enormous effect, especially considering that most children dying from disease are very young. Disease modelling work from Knight and colleagues on the effect of TB vaccines has shown that a vaccine with even a moderate efficacy of 60% would avert a total of 17 million TB cases by 2050, especially when targeting adolescents and adults [125]. This and other modelling studies [126,127] highlight the potential impact of interventions, even if efficacy is suboptimal.

**Table 5:** Interventions that might reduce the impact of CMV on TB progression from infection to disease.

Question	Knowledge gap (summary)	Potential study designs	Parameters and samples to be evaluated
Identifying interventions	- Identifying easy-to-implement interventions (e.g., parenting practices around handwashing and avoiding kissing, etc.)	- Systematic review of existing literature- Observational and randomised complex intervention studies in humans- Longitudinal cohorts from varying geographical areas and with different patient populations	Acute versus latent infection (CMV) - Viral detection: Whole blood (EDTA) for viral load (PCR), respiratory specimen, and others - Serology (plasma/serum): Quantitative IgG, IgM, and IgG avidity TB diagnostic workup: - TB microbiology - Immunoassays evaluating T-cell response
Evaluating interventions	- Impact of easy-to-implement intervention on CMV prevalence and its influence on TB epidemiology (e.g., parenting practices around handwashing, kissing, etc.) - Identifying which groups will benefit most from interventions and which will not - Impact of interventions (high/low efficacy) on TB epidemiology and life course of children (long-term morbidities such as lung outcome, reinfections, etc.) - Impact of reduced transmission in adolescents following CMV interventions	- Systematic review of existing literature - Observational and randomised intervention studies in humans - Longitudinal cohorts from varying geographical areas and with different patient populations - Correlates of protection studies in humans. - Modelling studies to quantify impact	Acute versus latent infection (CMV) - Viral detection: whole blood (EDTA) for viral load (PCR), respiratory specimen and others - Serology (plasma/serum): quantitative IgG, IgM, and IgG avidity TB diagnostic workup: - TB microbiology - Immunoassays evaluating T-cell response

CMV, cytomegalovirus; PCR, polymerase chain reaction; TB, tuberculosis.

Exploring TB and CMV infection and immune responses within TB and CMV vaccine trial cohorts could determine whether these (and other) coinfections effect efficacy or immunogenicity of novel vaccine candidates. Determining whether the CMV infection is a primary infection or reactivation might inform which CMV vaccine could be used. Again, to make maximal use of a well-characterised cohorts, collection and storage of a wide range of clinical samples for more basic research purposes should be incorporated.

## The way forward

Epidemiologic and immunologic evidence exists for an interaction between TB and CMV. Better data are required to confirm a direct link, to understand the scope of the relationship, and elucidate mechanisms of CMV–TB coinfection. Part of our poor understanding stems from inconsistent and nonstandardised definitions and testing processes. There is a need for head-to-head comparisons of the existing and new assays for diagnostic tests for both pathogens, particularly for CMV. As yet, there is no consensus on the sample type or assays to be used. If we could diagnose and classify these 2 diseases in a consistent way so that all researchers are speaking the same language, then shared biological pathways could be identified and appropriate interventions planned. Armed with potential drug therapies, vaccines, and sociobehavioural interventions, appropriate studies could be designed to evaluate them, with adequate sample size and clearly defined endpoints. Once the efficacy of interventions is established, implementation in LMIC settings will be challenging, but, increasingly, there is recognition that complex health interventions can be implemented in any context [128–130]. Good examples are the widespread delivery of infant vaccines and increasingly universal antiretroviral therapy access. The ambitious targets set by the World Health Organization in their End TB Strategy to reduce TB deaths by 95% and to cut new cases by 90% between 2015 and 2035 will only be met by addressing all aspects of TB, including the impact of coinfections such as CMV. The challenges for child and adolescent TB are substantial, and an improved understanding of the relationship between CMV and TB may be key to reducing morbidity and mortality in this age group.
